# Nutrition and Health – The Association between Eating Behavior and Various Health Parameters: A Matched Sample Study

**DOI:** 10.1371/journal.pone.0088278

**Published:** 2014-02-07

**Authors:** Nathalie T. Burkert, Johanna Muckenhuber, Franziska Großschädl, Éva Rásky, Wolfgang Freidl

**Affiliations:** Institute of Social Medicine and Epidemiology, Medical University Graz, Graz, Austria; Geisel School of Medicine at Dartmouth College, United States of America

## Abstract

Population-based studies have consistently shown that our diet has an influence on health. Therefore, the aim of our study was to analyze differences between different dietary habit groups in terms of health-related variables. The sample used for this cross-sectional study was taken from the Austrian Health Interview Survey AT-HIS 2006/07. In a first step, subjects were matched according to their age, sex, and socioeconomic status (SES). After matching, the total number of subjects included in the analysis was 1320 (N = 330 for each form of diet – vegetarian, carnivorous diet rich in fruits and vegetables, carnivorous diet less rich in meat, and carnivorous diet rich in meat). Analyses of variance were conducted controlling for lifestyle factors in the following domains: health (self-assessed health, impairment, number of chronic conditions, vascular risk), health care (medical treatment, vaccinations, preventive check-ups), and quality of life. In addition, differences concerning the presence of 18 chronic conditions were analyzed by means of Chi-square tests. Overall, 76.4% of all subjects were female. 40.0% of the individuals were younger than 30 years, 35.4% between 30 and 49 years, and 24.0% older than 50 years. 30.3% of the subjects had a low SES, 48.8% a middle one, and 20.9% had a high SES. Our results revealed that a vegetarian diet is related to a lower BMI and less frequent alcohol consumption. Moreover, our results showed that a vegetarian diet is associated with poorer health (higher incidences of cancer, allergies, and mental health disorders), a higher need for health care, and poorer quality of life. Therefore, public health programs are needed in order to reduce the health risk due to nutritional factors.

## Introduction

Our diet has an impact on our well-being and on our health. Studies have shown a vegetarian diet to be associated with a lower incidence of hypertension, cholesterol problems, some chronic degenerative diseases, coronary artery disease, type II diabetes, gallstones, stroke, and certain cancers [1]–[7]. A vegetarian diet is characterized by a low consumption of saturated fat and cholesterol, due to a higher intake of fruits, vegetables and whole-grain products [3], [4], [8]. Overall, vegetarians have a lower body mass index [1], [4], [5], [7], [9]–[12], a higher socioeconomic status [13], and better health behavior, i.e. they are more physically active, drink less alcohol, and smoke less [9], [13], [14]. On the other hand, the mental health effects of a vegetarian diet or a Mediterranean diet rich in fruits, vegetables, whole-grain products and fish are divergent [9], [15]. For example, Michalak et al. [16] report that a vegetarian diet is associated with an elevated prevalence of mental disorders. A poor meat intake has been shown to be associated with lower mortality rates and higher life expectancy [17], and a diet which allows small amounts of red meat, fish and dairy products seems to be associated with a reduced risk of coronary heart disease as well as type 2 diabetes [18]. Additionally, evidence concerning lower rates of cancer, colon diseases including colon cancer, abdominal complaints, and all-cause mortality is, however, inconsistent [5]–[7], [19]–[22].

Not only is the intake of certain nutrients, like red meat, associated with an increased health risk [18], [23]–[26], high-caloric intake also plays a crucial role [23], [27]. Moreover, there seems to be proof that lifestyle factors like physical activity may be more crucial in lowering disease rates than individual dietary habits [20], [28]–[29]. While, generally speaking, diets based on plants, like vegetarian diets, seem to be associated with a certain health benefit, a lower risk to contract certain chronic diseases [30], and the ability to improve health [31]–[32], restrictive and monotonous vegetarian diets include the risk of nutritional deficits [2], [18], [19], [30], [33]. Baines et al. [9] report that vegetarians take more medication than non-vegetarians.

To summarize, a number of studies have shown vegetarian diets and diets with poor meat intake to be associated with lower mortality rates for certain diseases. Research about the dietary habits in Austria is, however, rather sparse and mainly focused on genetic factors [33]–[36]. Therefore, the aim of this study was to investigate health differences between different dietary habit groups among Austrian adults.

## Methods

### Study Design and Study Population

The sample for this cross-sectional study was taken from the Austrian Health Interview Survey (AT-HIS) which ran from March 2006 to February 2007 [37]. The AT-HIS is a standardized survey which is conducted at regular intervals in Austria (currently every eight years). The subjects included in the survey form a representative sample of the Austrian population. They were chosen from the central population register and are distributed across the different geographic regions of Austria. The AT-HIS is part of the European Health Interview Survey (E-HIS; http://www.euhsid.org), an important high-quality survey. The interviews were conducted by free-lancers engaged by the Austrian Statistic Agency. To ensure that all interviews were conducted in the same way, interviewers had to participate in a training day where they were instructed on how to conduct the survey. Time measurement, non-response analyses, and analyses of error dialogs were performed in order to ensure consistency between interviewers. Additionally, all interviewers were supervised by field supervisors. Overall, 15474 individuals, aged 15 years and older, were questioned in computer-assisted personal interviews (CAPI; 54.7% female; response rate: 63.1%).

While 0.2% of the interviewees were pure vegetarians (57.7% female), 0.8% reported to be vegetarians consuming milk and eggs (77.3% female), and 1.2% to be vegetarians consuming fish and/or eggs and milk (76.7% female). 23.6% reported to combine a carnivorous diet with lots of fruits and vegetables (67.2% female), 48.5% to eat a carnivorous diet less rich in meat (60.8% female), and 25.7% a carnivorous diet rich in meat (30.1% female). Since the three vegetarian diet groups included a rather small number of persons (N = 343), they were analyzed as one dietary habit group. Moreover, since the vegetarian group was the smallest, we decided to match each of the vegetarians (1) with an individual of each other dietary habit group (carnivorous diet rich in fruits and vegetables (2), carnivorous diet less rich in meat (3) and a carnivorous rich in meat (4)).

### Matching Process

In a first step, subjects consuming a vegetarian diet were identified (N = 343). All vegetarians were categorized according to their sex, age (in age-groups spanning 5 years, e.g. 20- to 24-year-olds), and socioeconomic status (SES). Each such vegetarian was then matched with one subject consuming a carnivorous diet rich in fruits and vegetables, one individual eating a carnivorous diet less rich in meat, and one subject consuming a carnivorous diet rich in meat. Only 96.2% of the vegetarians were included in the analyses, since not all of them corresponded to a subject of the same sex, age, and SES from a different dietary habit group. Therefore, the total number of analyzed subjects was 1320 (comprising 330 vegetarians, 330 subjects consuming a carnivorous diet rich in fruits and vegetables, 330 individuals eating a carnivorous diet less rich in meat, and 330 subjects consuming a carnivorous diet rich in meat). Each dietary habit group was set-up according to the demographic characteristics shown in Table 1.

**Table 1:** Data definition and structure for each of the four dietary habit groups.

Age		Sex		TOTAL	
		men	women		
	SES	N	N	N	%
**15–19**	Low	3	22	25	7.6%
	Middle	9	19	28	8.5%
	High	0	1	1	0.3%
**20–29**	Low	4	11	15	4.5%
	Middle	10	35	45	13.6%
	High	5	13	18	5.4%
**30–39**	Low	3	10	13	3.9%
	Middle	6	23	29	8.8%
	High	6	11	17	5.2%
**40–49**	Low	4	6	10	3.0%
	Middle	4	20	24	7.3%
	High	8	16	24	7.3%
**50–59**	Low	3	7	10	3.0%
	Middle	3	11	14	4.2%
	High	1	6	7	2.1%
**60–69**	Low	3	7	10	3.0%
	Middle	1	14	15	4.5%
	High	0	2	2	0.6%
**70–79**	Low	4	8	12	3.6%
	Middle	1	2	3	0.9%
	High	0	0	0	0.0%
**80+**	Low	0	5	5	1.5%
	Middle	0	3	3	0.9%
	High	0	0	0	0.0%
**TOTAL**	Low	24	76	100	30.3%
	Middle	34	127	161	48.8%
	High	20	49	69	20.9%
**TOTAL**		**78**	**252**	**330**	**100.0%**

*Note.* Data source: Austrian Health Interview Survey (AT-HIS) 2006/07. N = number of subjects. Analyses were calculated with subjects matched according to their age, sex, and socio-economic status. Each dietary habit group consisted of 330 subjects with the above shown demographic characteristics (330 vegetarians, 330 subjects consuming a carnivorous diet rich in fruits and vegetables, 330 individuals eating a carnivorous diet less rich in meat, and 330 subjects consuming a carnivorous diet rich in meat; N = 1320). SES: A low SES corresponds a SES score between ≥3 and ≤6, a middle SES between >6 and ≤10, and a high SES a score between >10 and ≤15.

### Ethical Approval

The study was carried out in compliance with the principles laid down in the Helsinki Declaration. No minors or children were included in the study. Verbal informed consent was obtained from all subjects, witnessed, and formally recorded. The Ethics Committee of the Medical University of Graz approved the consent procedure as well as the conductance of this study (EK-number: 24–288 ex11/12).

### Variables and Measurements

Face-to-face interviews were conducted by questioning the subjects about their socio-demographic characteristics, health-related behavior, diseases, medical treatments, and also psychological aspects.

The independent variable in this study was the dietary habit of individuals. Concerning eating behavior, the respondents were given a list of six different dietary habits and asked which one describes their eating behavior best (1 = vegan, 2 = vegetarian eating milk/eggs, 3 = vegetarian eating fish and/or milk/eggs, 4 = carnivorous diet rich in fruits and vegetables, 5 = carnivorous diet less rich in meat, 6 = carnivorous diet rich in meat). Participants described their dietary habit, without interviewers giving a clear definition of the various eating categories. Since, overall, only 2.2% of all participants consumed a vegetarian diet, these individuals were analyzed as one dietary habit group. We created a scale that would reflect the animal fat intake for each dietary habit (1 = vegetarian diet, 2 = carnivorous diet rich in fruits and vegetables, 3 = carnivorous diet less rich in meat, 4 = carnivorous diet rich in meat).

Since age, sex, and the socioeconomic background of subjects all have an influence on health [38]–[41], we matched the subjects according to these variables in order to control for their influence. The SES of the subjects (ranging between 3 and 15) was calculated using the following variables: net equivalent income, level of education, and occupation. Net equivalent income was calculated based on an equivalence scale provided by the OECD [42], and divided by quintiles. Level of education was measured by an ordinal variable, distinguishing between (1) basic education (up to 15 years of age), (2) apprenticeship/vocational school, (3) secondary education without diploma, (4) secondary education with diploma, and (5) university education. The occupation of the subjects was differentiated into the following five levels: (1) unskilled worker, (2) apprentice/skilled worker, (3) self-employed/middle job, (4) qualified job/academic, (5) executive position. To verify the combination of variables that served to calculate the SES, correlations with the different variables were calculated. They ranged between r = .70 and r = .80.

The body mass index (BMI) and lifestyle factors (physical activity, smoking, and alcohol consumption) were included as covariates in all analyses. The BMI was calculated by dividing the weight of a person in kilograms by the square of their height in meters (kg/m^2^) [43]. Physical exercise was measured using the short version of the International Physical Activity Questionnaire (IPAQ), a self-reported instrument, which asks for an estimate of the total weekly physical activity (walking, moderate- and vigorous-intensity activity) performed during the last week. The short version of the IPAQ does not discriminate between leisure-time and non-leisure time physical activity. The total MET score was calculated by weighting the reported minutes per week within each activity by a MET energy expenditure estimate that was assigned to each category [44]. Smoking behavior was measured as the number of cigarettes smoked per day. Alcohol consumption was surveyed as the number of days on which alcohol was consumed during the last 28 days.

The dependent variables focusing on ill-health included self-perceived health, ranging from 1 (very good) to 5 (very bad), and impairment to health, ranging from 1 (very impaired) to 3 (not impaired). We further assessed the presence of 18 specific chronic conditions (asthma, allergies, diabetes, cataract, tinnitus, hypertension, cardiac infarction, apoplectic stroke, bronchitis, arthritis, sacrospinal complaints, osteoporosis, urinary incontinence, gastric or intestinal ulcer, cancer, migraine, mental illness (anxiety disorder or depression), and any other chronic condition). Each condition was coded as present (1) or absent (0). We calculated a total frequency score by summing up the chronic conditions present (0–18, sum index). Additionally, a vascular risk score was calculated by summing up the variables “hypertension”, “enhanced blood cholesterol level”, “diabetes”, and “smoking” (0–4, sum index). Each variable was coded as present (1) or absent (0).

A dependent variable concerning health care was created as the sum index of the number of doctors consulted in the last 12 months (0–8, sum index). Each of the 8 medical treatments (general practitioner, gynecologist, urologist, dermatologist, ophthalmologist, internist, orthopedist, and ENT physician) was coded as “consulted” (1) or “not consulted” (0). The number of vaccinations was analyzed by calculating a sum index combining 8 different vaccinations (influenza, tetanus, diphtheria, polio, FSME, pneumococci, hepatitis A and B; 0–8, sum index). Each vaccination was coded as present (1) or absent (0). In addition, preventive health care was analyzed by calculating a sum index of the variables “preventive check-ups”, “mammography”, “prostate gland check-up”, and “Papanicolaou test” (0–4, sum index). Each variable was coded as present (1) or absent (0).

The dependent variable concerning quality of life was measured using the short version of the WHOQOL (WHOQOL-BREF) [45]. Four domain scores (physical health, psychological health, social relationships, and environment) were calculated. These domain scores ranged between 4 and 20.

### Statistical Analysis

In a first step subjects with different dietary habits (vegetarian, carnivorous diet rich in fruits and vegetables, carnivorous diet less rich in meat, carnivorous diet rich in meat) were matched according to their sex, age, and SES. Differences in lifestyle factors (BMI, total MET score, number of cigarettes smoked per day, and alcohol consumption in the last four weeks) between the different dietary habit groups were calculated by multivariate analysis of variance.

In order to analyze the differences between the dietary habit groups, multivariate analyses of variance were calculated for the three domains: (1) health (self-reported health, impairment due to health problems, number of chronic conditions, vascular risk), (2) health care (number of visits to the doctor, number of vaccinations, number of used preventive care offers), and (3) quality of life (physical and psychological health, social relationships, and environment). To address the bias of lifestyle factors impacting health, analyses of variance were calculated, controlling for the aforementioned lifestyle variables (BMI, physical activity, smoking behavior, and alcohol consumption).

In the domain of “health”, the two variables “self-reported health” and “impairment due to health problems” were originally assessed using an ordinal scale. Therefore, we controlled the results using non-parametric tests (Kruskal Wallis Test). Since the results were the same, only results of the analyses of variance are reported.

In addition, Chi-square tests were calculated for the aforementioned 18 chronic conditions in order to establish which one occurs significantly more often, depending on the form of nutrition. *p*-values <.*050* were considered as statistically significant. All analyses were calculated using IBM SPSS software (version 20.0) for Windows.

## Results

### Participant Characteristics and Lifestyle Differences between the Dietary Habit Groups

In total, we analyzed the data of 1320 individuals (330 in each dietary habit group). Each dietary habit group was set-up according to the demographic characteristics shown in Table 1. Overall, 23.6% of all subjects were male and 76.4% female. 40.0% of the individuals were younger than 30 years, 17.8% between 30 and 39 years, 17.6% between 40 and 49 years, 9.4% between 50 and 59 years, 8.4% between 60 and 69 years, 4.4% between 70 and 79 years, and 2.4% than 80 years or older. 30.3% of the subjects had a low SES (they had an SES score of ≤6), 48.8% a middle one (SES between >6 and ≤10), and 20.9% had a high SES (SES>10).

Our multivariate analysis regarding lifestyle showed a significant main effect for the dietary habit of individuals (p = .000), showing that the different dietary habit groups differ in their overall health behavior. However, results of the univariate analyses showed that the dietary habit groups only differ concerning their BMI and their alcohol consumption.

Concerning BMI: vegetarians have the lowest mean BMI (M = 22.9), followed by subjects eating a carnivorous diet less rich in meat (M = 23.4), rich in fruits and vegetables (M = 23.5), and rich in meat (M = 24.9). Heavy meat eaters differ significantly from all other groups in terms of their BMI (p = .000).

Concerning physical exercise: no significant difference was found in the total MET score between the various dietary habit groups (p = .631).

Concerning smoking behavior: the number of cigarettes smoked per day did not differ between the various dietary habit groups (p = .302).

Concerning alcohol consumption: Subjects following a vegetarian diet (M = 2.6 days in the last 28 days) or a carnivorous diet rich in fruits and vegetables (M = 3.0 days) consume alcohol significantly less frequently than those eating a carnivorous diet less rich in meat (M = 4.4 days) or rich in meat (M = 4.8 days; p = .000).

### Health Differences between the Dietary Habit Groups

In the domain of health, the multivariate analysis of variance showed a significant main effect for the dietary habit of individuals (p = .000). Overall, vegetarians are in a poorer state of health compared to the other dietary habit groups. Concerning self-reported health, vegetarians differ significantly from each of the other groups, toward poorer health (p = 000). Moreover, these subjects report higher levels of impairment from disorders (p = .002). Vegetarians additionally report more chronic diseases than those eating a carnivorous diet less rich in meat (p = .000; Table 2). Significantly more vegetarians suffer from allergies, cancer, and mental health ailments (anxiety, or depression) than the other dietary habit groups (Table 3). Subjects who eat a carnivorous diet rich in meat more often report urinary incontinence (p = .023). No differences between individuals consuming different forms of diet were found regarding their vascular risk (p = .150; Table 2).

**Table 2:** Differences in health and health care between the different dietary habit groups.

	vegetarian N = 330		carnivorous diet rich in fruits and vegetables N = 330		carnivorous diet lessrich in meat N = 330		carnivorous diet rich in meat N = 330		
Measure	M	SD	M	SD	M	SD	M	SD	p-value
**Health**									**.000** ^3^
Self-reported health generally^1^	1.78	0.94	1.50	0.90	1.46	0.87	1.57	0.87	**.000** ^4^
Impairment^2^	2.62	0.66	2.71	0.62	2.72	0.59	2.73	0.61	**.002** ^4^
Chronic conditions^1^	1.29	1.60	1.00	1.64	0.92	1.29	1.03	1.60	**.000** ^4^
Vascular risk^1^	2.02	0.74	1.96	0.75	2.01	0.71	1.98	0.73	**.150** ^4^
**Health care**									**.000** ^3^
Medical treatments (number of visits to the doctor)^1^	1.69	1.33	1.68	1.27	1.43	1.02	1.60	1.22	**.003** ^4^
Number of vaccinations^2^	3.22	2.28	3.65	2.15	3.59	2.02	3.61	2.13	**.005** ^4^
Number of preventive healthcare offers used^2^	1.25	1.07	1.39	1.07	1.29	1.07	1.26	1.05	**.033** ^4^

*Note.* Data source: Austrian Health Interview Survey (AT-HIS) 2006/07. M = mean, SD = standard deviation, N = number of subjects, p = probability.

1 a higher score means worse results, ^2^a higher score means better results,^ 3^multivariate test result, ^4^result of univariate comparison. Analyses were calculated with subjects matched according to their age, sex, and socio-economic status controlling for BMI, physical activity (total MET score), smoking behavior (number of cigarettes per day), and alcohol consumption (number of days on which alcohol was consumed during the last 28 days) (N = 1320).

**Table 3:** Differences in suffering from various chronic conditions between the different dietary habit groups.

Chronic condition	Vegetarian	Carnivorous diet rich in fruits and vegetables	Carnivorous diet lessrich in meat	Carnivorous dietrich in meat	p-value (χ^2^)
Asthma	4.8%	3.3%	3.9%	4.5%	.772
Allergies	**30.6%**	**18.2%**	**20.3%**	**16.7%**	**.000**
Diabetes	2.7%	4.2%	2.4%	2.4%	.455
Cataract	4.2%	3.0%	3.3%	1.8%	.348
Tinnitus	4.8%	4.8%	4.8%	3.6%	.840
Hypertension	11.5%	10.6%	12.4%	15.5%	.260
Cardiac infarction	1.5%	1.5%	0.9%	0.6%	.610
Apoplectic stroke	1.2%	1.8%	1.5%	1.8%	.610
Bronchitis	3.9%	3.6%	2.4%	3.0%	.701
Arthritis	8.5%	7.6%	8.8%	10.3%	.662
Sacrospinal complaints	26.7%	24.8%	18.2%	23.9%	.060
Osteoporosis	6.4%	4.8%	3.6%	5.8%	.415
Urinary incontinence	**2.1%**	**3.9%**	**2.7%**	**6.4%**	**.023**
Gastric or intestinal ulcer	4.2%	4.2%	1.5%	3.6%	.169
Cancer	**4.8%**	**3.3%**	**1.2%**	**1.8%**	**.022**
Migraine	15.8%	11.8%	9.1%	12.1%	.074
Mental illness (anxietydisorder or depression)	**9.4%**	**4.8%**	**5.8%**	**4.5%**	**.036**
Any other chronic conditions	8.8%	5.5%	5.8%	6.7%	.308

*Note.* Data source: Austrian Health Interview Survey (AT-HIS) 2006/07. Percentage of subjects suffering from the different chronic conditions. p (χ^2^): probability value of Chi-Square-Test. Analyses were calculated with subjects matched according to their age, sex, and socio-economic status (N = 1320).

### Differences in Health Care between the Dietary Habit Groups

Our multivariate analysis regarding health care has shown a significant main effect for dietary habits (p = .000) and confirmed that, overall, subjects with a lower animal fat intake demonstrate worse health care practices. Vegetarians and subjects eating a carnivorous diet rich in fruits and vegetables consult doctors more often than those eating a carnivorous diet less rich in meat (p = .003). Moreover, vegetarians are vaccinated less often than all other dietary habit groups (p = .005) and make use of preventive check-ups less frequently than subjects eating a carnivorous diet rich in fruits and vegetables (p = .033; Table 2).

### Differences in Quality of Life between the Dietary Habit Groups

Regarding quality of life, the main effect of the multivariate analysis of variance showed no significant difference between the dietary habit groups (p = .291). The results obtained in the univariate analyses of variance, however, revealed that vegetarians have a lower quality of life in the domains of “physical health” (p = .026) and “environment” (p = .037) than subjects consuming a carnivorous diet less rich in meat. Moreover, vegetarians have a lower quality of life regarding “social relationships” than individuals eating a carnivorous diet rich in fruits and vegetables, or those with a carnivorous diet less rich in meat (p = .043). All results are shown in Table 4.

**Table 4:** Differences in quality of life between the different dietary habit groups.

	vegetarian N = 330		carnivorous diet rich in fruits and vegetables N = 330		carnivorous diet less rich in meat N = 330		carnivorous diet rich in meat N = 330		
Measure	M	SD	M	SD	M	SD	M	SD	p-value
**Quality of life**									.291^2^
WHOQOL physical health^1^	17.16	3.26	17.52	2.95	17.68	2.90	17.40	2.86	**.026** ^3^
WHOQOL psychological health^1^	16.50	2.74	16.75	2.42	16.88	2.33	16.66	2.24	.116^3^
WHOQOL social relationships^1^	16.59	2.82	16.99	2.49	16.96	2.38	16.88	2.63	**.043** ^3^
WHOQOL environment^1^	16.16	2.26	16.44	2.11	16.56	1.98	16.45	2.08	**.037** ^3^

*Note.* Data source: Austrian Health Interview Survey (AT-HIS) 2006/07. M = mean. SD = standard deviation. N = number of subjects. p = probability.

1 a higher score means better results, ^2^multivariate test result, ^3^result of univariate comparison. Analyses were calculated with subjects matched according to their age, sex, and socio-economic status controlling for BMI, physical activity (total MET score), smoking behavior (number of cigarettes per day), and alcohol consumption (number of days on which alcohol was consumed during the last 28 days) (N = 1320).

## Discussion

Overall, our findings reveal that vegetarians report poorer health, follow medical treatment more frequently, have worse preventive health care practices, and have a lower quality of life. Concerning the variable “eating behavior”, we tried to generate a variable that would reflect the animal fat intake (1 = vegetarian, 2 = carnivorous diet rich in fruits and vegetables, 3 = carnivorous diet less rich in meat, 4 = carnivorous diet rich in meat). The mean BMI of subjects is coupled in nearly linear progression with the amount of animal fat intake. This is in line with previous studies showing vegetarians to have a lower body mass index [1], [4], [5], [7], [9]–[12].

Our results have shown that vegetarians report chronic conditions and poorer subjective health more frequently. This might indicate that the vegetarians in our study consume this form of diet as a consequence of their disorders, since a vegetarian diet is often recommended as a method to manage weight [10] and health [46]. Unfortunately, food intake was not measured in more detail, e.g. caloric intake was not covered. Hence, further studies will be necessary to analyze health and its relationship with different forms of dietary habits in more detail.

When analyzing the frequency of chronic diseases, we found significantly higher cancer incidence rates in vegetarians than in subjects with other dietary habits. This is in line with previous findings, reporting that evidence about cancer rates, abdominal complaints, and all-cause mortality in vegetarians is rather inconsistent [5]–[7], [19]–[22]. The higher cancer incidence in vegetarians in our study might be a coincidence, and is possibly related to factors other than the general amount of animal fat intake, such as health-conscious behavior, since no differences were found regarding smoking behavior and physical activity in Austrian adults as reported in other studies for other countries [9], [13], [14]. Therefore, further studies will be required in Austria in order to analyze the incidence of different types of cancer and their association with nutritional factors in more depth.

Several studies have shown the mental health effects of a vegetarian diet to be divergent [9], [15], [16]. Vegetarians in our study suffer significantly more often from anxiety disorder and/or depression. Additionally, they have a poorer quality of life in terms of physical health, social relationships, and environmental factors.

Moreover, the use of health care differs significantly between the dietary habit groups in our study. Vegetarians need more medical treatment than subjects following another form of diet. However, this might be due to the number of chronic conditions, which is higher in subjects with a vegetarian diet.

Among the **strengths** of our study are: the large sample size, the matching according to age, sex, and socioeconomic background, and the standardized measurement of all variables. Other strengths of our study include considering the influence of weight and lifestyle factors on health, e.g. physical exercise and smoking behavior.

Potential **limitations** of our results are due to the fact that the survey was based on cross-sectional data. Therefore, no statements can be made whether the poorer health in vegetarians in our study is caused by their dietary habit or if they consume this form of diet due to their poorer health status. We cannot state whether a causal relationship exists, but describe ascertained associations. Moreover, we cannot give any information regarding the long-term consequences of consuming a special diet nor concerning mortality rates. Thus, further longitudinal studies will be required to substantiate our results. Further limitations include the measurement of dietary habits as a self-reported variable and the fact that subjects were asked how they would describe their eating behavior, without giving them a clear definition of the various dietary habit groups. However, a significant association between the dietary habit of individuals and their weight and drinking behavior is indicative for the validity of the variable. Another limitation concerns the lack of detailed information regarding nutritional components (e.g. the amount of carbohydrates, cholesterol, or fatty acids consumed). Therefore, more in-depth studies about nutritional habits and their effects on health are required among Austrian adults. Further studies should e.g. investigate the influence of the various dietary habits on the incidence of different cancer types. To our knowledge this is the first study ever in Austria to analyze differences in terms of dietary habits and their impact on health. We admit that the large number of participants made it necessary to keep the questions simple, in order to cover the large sample. Overall, we feel that our results are of specific interest and contribute to extant scientific knowledge, notwithstanding some limitations regarding causes and effects.

## Conclusions

Our study has shown that Austrian adults who consume a vegetarian diet are less healthy (in terms of cancer, allergies, and mental health disorders), have a lower quality of life, and also require more medical treatment. Therefore, a continued strong public health program for Austria is required in order to reduce the health risk due to nutritional factors. Moreover, our results emphasize the necessity of further studies in Austria, for a more in-depth analysis of the health effects of different dietary habits.
